# Radio Frequency Ablation in Drug Resistant Chemotherapy-induced Peripheral Neuropathy: A Case Report and Review of Literature

**DOI:** 10.4103/0973-1075.63135

**Published:** 2010

**Authors:** Naveen Yadav, Frenny Ann Philip, Vikas Gogia, Prakash Choudhary, Shiv Pratap Sing Rana, Seema Mishra, Sushma Bhatnagar

**Affiliations:** Dr. B. R. Ambedkar, Institute Rotary Cancer Hospital, All India Institute of Medical Sciences, Ansari Nagar, New Delhi-110 029, India

**Keywords:** Chemotherapy-induced neuropathy, Median nerve, Pulse radiofrequency, Ulnar nerve

## Abstract

Chemotherapy-induced peripheral neuropathy (CIPN) is a frequently encountered complication. It can result from a host of agents. Various modalities of treatment have been advocated, of which a novel method is radio frequency ablation. A 63-year-old male, a case of carcinoma prostrate with bone metastases, presented with tingling and numbness in right upper limb. He was given morphine, gabapentin and later switched to pregabalin, but medications provided only minor relief. Initially he was given stellate ganglion block, then radiofrequency ablation of dorsal root ganglion was done, but it failed to provide complete relief. Pulsed radiofrequency ablation (PRF) was then done for 90 seconds; two cycles each in both ulnar and median nerve. After the procedure the patient showed improvement in symptoms within four to five hours and 80% relief in symptoms. We conclude that PRF can be used for the treatment of drug resistant CIPN.

## INTRODUCTION

Chemotherapy-induced peripheral neuropathy (CIPN) is a common side effect observed following exposure of patients to the vinca-alkaloids, the taxanes, the platinum derived compounds, suramin, thalidomide and most recently also associated with bortezomib therapy. The onset of chemo-neuropathy is generally early in treatment, between the first and third cycle, with the peak in severity occurring approximately three months into therapy.[1,2] Patients usually present with tingling numbness and pain in glove and stocking pattern.

The time course for resolution of CIPN is highly variable. Several pharmacological agents have been used in the prevention and treatment of established CIPN. They are tricyclic antidepressant, gabapentin, pregabalin, lamotrigine, topical combination of baclofen, amitrypline and ketamine and Acetyl-L-Carnitine. The RF lesioning technique involves placement of an insulated needle with an active tip in the vicinity of a nerve or ganglion.[3] Two types of RF lesioning are used clinically: continuous radiofrequency (CRF) and pulsed radio frequency (PRF). PRF use brief pulses of high voltage of electric current. Numerous case reports and case series of use of PRF in peripheral nerves and ganglions for treatment of chronic pain can be found in the literature.

We present the case of a 63-year-old male of carcinoma prostrate with multiple bone metastases who had received chemotherapy with docitaxel.

## CASE REPORT

A 63-year-old male with carcinoma prostrate was being treated in our hospital. The patient had developed multiple bony metastases in acetabulam, left ischium, femur head, spine, ribs, pelvis and skull. Patient had undergone bilateral orchidectomy and was on biclutamide, an antiandrogen.

Patient was given chemotherapy with docitaxel and prednisolone after surgery along with palliative radiotherapy to hemipelvis. Three months after the surgery and completion of chemotherapy, the patient started complaining of pain in the right palm and tingling sensation in right hand. There was also numbness present in the distribution of right arm, forearm and hand. The patient was investigated and a cervical X-ray showed C5-C6 block vertebrae and C2-C3 fusion (cervical spondylosis). Stellate ganglion block was performed but no significant relief was noted. The patient was put on morphine and gabapentin.

The patient initially had some relief with oral medications. But soon higher doses of morphine were needed for pain control and radiofrequency ablation of dorsal root ganglion (cervical) was planned. The patient had good relief of arm and forearm pain with mild pain persisting in right palm. The pain gradually increased in severity after eight months, though earlier the patient was stable on a dose of morphine and pregabalin. Cervical epidural steroid was tried, but it gave only transient relief. Pain and numbness in the right hand increased, with patient needing increased morphine doses.

A differential diagnosis of carpal tunnel syndrome was considered. Nerve conduction velocity study was done. Motor nerve conduction was normal in all tested nerves. Sensory nerve conduction study revealed latency difference of 0.6 milliseconds on the right side and 0.3 milliseconds on the left side, between the median and ulnar nerve. Nerve conduction studies were conclusive of Grade 1 carpal tunnel syndrome on right side. Nerve conduction study showed that carpal tunnel syndrome was unlikely to be the causative agent.

Pulsed radiofrequency of the median and ulnar nerve was considered. Informed consent was obtained. Intravenous access was secured. The patient was made supine and right wrist was extended. Under full aseptic precautions, using landmark technique and nerve stimulator guidance, the pulse radiofrequency ablation was done for 90 seconds two cycles in both ulnar and median nerve. The patient started showing relief in the symptoms within two to four hours. On the next day, the patient reported 40% relief in pain. Within two days patient reported relief of 90% in pain. The dose of morphine was stopped and patient was discharged with no pain medications.

## DISCUSSION

Cancer patients suffer from numerous symptoms including fatigue, depression, sleep disturbance and pain that often co-occur and interact to magnify their respective impact. Disease and treatment related pain is a major cause of treatment failure. Pain is not only associated with active disease, but is also a common symptom that persists in cancer survivors. Cancer related pain has multiple mechanisms. The direct invasion by tumors into bone, soft tissues and viscera produces nociceptive, somatic and visceral pain, whereas tumor invasion and compression of the spinal cord and nerves causes neuropathic pain. Brachial and lumbosacral plexopathies are relatively common in patients with lung, breast and abdominal malignancies.

CIPN is a common side effect observed post exposure to vinca-alkaloids, taxanes, platinum derived compounds, suramin, thalidomide and most recently with bortezomib therapy. It appears that onset and severity depends on a variety of factors including concomitant medical conditions such as diabetes, alcoholism and paraneoplastic sensory neuropathy.

The onset of chemo-neuropathy is generally early in treatment, between the first and third cycle, with the peak in severity occurring approximately three months into therapy.[1,2] Onset is correlated to the magnitude of treatment dose. Patients receiving a dose of 500 mg/m^2^ paclitaxel or higher developed paraesthesias on average 3.3 days after treatment and severity of symptoms peaked at 10 days after the infusion.[3] Lower dose chemotherapy is often associated with a delayed- onset of paresthesias and dysesthesia, known as coasting effect, which occurs two to four months after chemotherapy.[4–5] The clinical presentation of the neuropathy is remarkably constant for all classes of chemotherapy drugs. Symptoms characteristically appear in a distal stocking- and-glove pattern.[2,6] Symptoms are described as numbness and tingling by more than 90% of patients and as overtly painful in 26% of cases.[2] Progression from numbness and tingling to pain may reflect an initial involvement of large myelinated fibers followed by further damage to small unmyelinated fibers.

Clinical examination of chemo neuropathy patients reveals an early loss of distal deep tendon reflexes followed by involvement of more proximal reflexes in a dose dependent manner. Ankle reflexes are decreased or absent in more than 95% of patients receiving paclitaxel and vincristine. Quantative sensory testing in subjects with chronic vincristine, paclitaxel, cisplatin or bortezomib induced peripheral neuropathy reveals many shared features as well as subtle disturbances. Patients with each type of neuropathy show increased touch sensation in areas directly affected by sensory disturbances, including the fingertips, palms, toes and soles of the feet, but also in volar skin outside the area of patient’s complaint. These changes likely underlie the impairment of daily activities noted in chemo neuropathy patients such as writing, buttoning clothes and handling objects.[2] Nerve biopsies from patients with paclitaxel and cisplatin peripheral neuropathy were remarkable for a length dependent loss of large fibers, axonal atrophy and secondary demyelination. The time course for resolution of chemotherapy induced peripheral neuropathy is highly variable. Neuropathic symptoms were present for a mean duration of 14.9 months in a group of patients with persistent paclitaxel-related pain.[2]

Compared to other neuropathies or neuropathic pain syndromes, CIPN resembles diabetic neuropathy with similar glove and stocking distribution and other characteristics, such as pain, paraesthesias, and dysaesthesias. However, treatments for diabetic neuropathies are not necessarily helpful for preventing or treating neuropathies associated with chemotherapy. The agents used in the prevention of CIPN are calcium and magnesium infusion, vitamin E, glutamine, glutathione, N-acetyl cysteine and anti-epileptic drugs like carbamazeipne. There are several pharmacological treatments available for established CIPN like tricyclic antidepressant, gabapentin, pregabalin, lamotrigine, topical combination of baclofen, amitryplineand ketamine and acetyl-L-carnitine.

The use of CRF treatment has been well-accepted in the realm of pain management practices, yet there remains a diverse body of conflicting literature that calls into question, not only its mode of action, but also its effectiveness.[7–8] Several studies failed to support nervous ablation, if actually occurring at all, as being responsible for the pain relief. In a recent report, Windsor reported that up to six CRF applications (70 degree C, 80 seconds) were necessary to sufficiently interrupt sensory neurotransmission in lumbar medial branch nerves in order to double the amount of 50 Hz current necessary to transmit the sensory impulse. This evidence strongly suggests that the beneficial effects of CRF treatment are independent of tissue destruction. Van Kleef *et al*. demonstrated that the pain relief produced by CRF of a dorsal root ganglion long outlasted the clinical evidence of denervation.[9] Slappendel *et al*. demonstrated equal efficacy of CRF and PRF treatments.[10] It has become more apparent that the generation of the electromagnetic field created by the RF current alters C fiber transmission by possibly altering sodium channel activity with associated changes in c-fos production in the dorsal horn. Pioneers in RF technology and treatment have even stated as much: “Eventually all this resulted in the suspicion that our assumptions might have been wrong and that heat might not be the element causing the clinical effect of a RF lesion”. Moreover, the risk of neuritis due to nervous tissue destruction is greatly reduced or eliminated with PRF.[11] As there was no obvious nervous tissue destruction with its attendant potential for painful neuritis, the “stun phase” was presumed to be responsible for the immediate relief of discomfort and was consistent with previously reported work. Thus, PRF, unlike CRF, can be and is used to treat peripheral nerves for various painful maladies.[11]

It is important to note the divergent techniques used in the two types of application. With CRF, electrode placement is directed parallel to the nerve, while with PRF, as most of the current is discharged through the electrode tip, the electrode is positioned perpendicular to the targeted structure. Also, as surrounding tissue fluids are felt to represent a “heat sink”, high tissue impedance is desired with CRF treatment. As a low tissue resistance allows more PRF current to be administered, efforts are made to maintain low tissue impedance during these applications. Moreover, when comparing RF studies, there is wide variability in the methodology used. For example, the proximity of the electrode to the targeted structure, as reflected by sensory stimulation parameters, is often accepted to be of any value less than 1 Volt.

The RF lesioning technique involves placement of an insulated needle with an active tip in the vicinity of a nerve or ganglion.[12] A grounded electrode is passed through the cannula and RF current is emitted at the tip of the needle.[13] There are two types of RF lesioning that are used clinically: CRF and PRF. CRF is the conventional method which uses a constant output of high frequency current and produces temperature >45 degree Celsius.[3] The heat production associated with this technique is neuroablative. Alternatively, PRF uses brief pulses of high voltage of electric current. Pauses between the pulses allow heat to dissipate and thus less nerve destruction occurs. The temperature with PRF does not exceed 45 degree Celsius. The exact mechanism of action of PRF is unknown.[12] However, the observation that sensory loss was transient while pain relief was of much longer duration lead to the hypothesis that temperature was not the only mechanism of action responsible for changes in pain perception. It has been proposed that PRF may act by modulating pain perception rather than directly destroying neural tissues.[14]

Numerous case reports and case series of PRF of numerous peripheral nerves and ganglions for treatment of chronic pain can be found in the literature.[13–18] Beneficial clinical results are found after PRF of the obturator nerve, femoral nerve, medial and lateral branches of the dorsal horn, stellate ganglion, supraclavicular nerve, S1 nerve root, the gasserian ganglion, glossopharyngeal nerve, sphenopalatine ganglion, ilioinguinal nerve, iliohypogastric nerve, genitofemoral nerve, supraorbital nerve, frontal nerve, and lateral femoral cutaneous nerve. No adverse effects of PRF were reported in any of these cases. Effects of pain relief appear to be variable, ranging from 2 to > 30 months with a mean duration of 9.2 months in one case series of PRF of the cervical DRG. An additional benefit of PRF is that the procedure can be repeated if pain recurs because minimal tissue has been destroyed.[18]

PRF is minimally invasive, well tolerated, and lacks potential adverse effects associated with high temperatures and thus holds promise in patients with chronic neuralgic pain that is refractory to conservative therapies.
